# Healthy eating and lifestyle in pregnancy (HELP): a protocol for a cluster randomised trial to evaluate the effectiveness of a weight management intervention in pregnancy

**DOI:** 10.1186/1471-2458-14-439

**Published:** 2014-05-10

**Authors:** Elinor John, Dunla M Cassidy, Rebecca Playle, Karen Jewell, David Cohen, Donna Duncan, Robert G Newcombe, Monica Busse, Eleri Owen-Jones, Nefyn Williams, Mirella Longo, Amanda Avery, Sharon A Simpson

**Affiliations:** 1 South East Wales Trials Unit, School of Medicine, Cardiff University, Neuadd Meirionnydd, Heath Park, Cardiff CF14 4YS, UK; 2 Faculty of Health Sport and Science, University of South Wales, Pontypridd CF37 1DL, UK; 3 Department of Primary Care and Public Health, School of Medicine, Cardiff University, Neuadd Meirionnydd, Heath Park, Cardiff CF14 4YS, UK; 4 School of Healthcare Sciences, Cardiff University, Ty Dewi Sant, Heath Park, Cardiff CF14 4XN, UK; 5 Schools of Health Care Sciences and Medical Sciences, Bangor University, Wrexham Technology Park, Wrexham LL13 7YP, UK; 6 School of Biosciences, University of Nottingham, Sutton Bonnington Campus, Leicestershire LE12 5RD, UK

**Keywords:** Study protocol, Pregnancy, Obesity, Complex intervention, Randomised controlled trial, Diet, Physical activity

## Abstract

**Background:**

Approximately 1 in 5 pregnant women in the United Kingdom are obese. In addition to being associated generally with poor health, obesity is known to be a contributing factor to pregnancy and birth complications and the retention of gestational weight can lead to long term obesity.

This paper describes the protocol for a cluster randomised trial to evaluate whether a weight management intervention for obese pregnant women is effective in reducing women’s Body Mass Index at 12 months following birth.

**Methods/design:**

The study is a cluster randomised controlled trial involving 20 maternity units across England and Wales. The units will be randomised, 10 to the intervention group and 10 to the control group. 570 pregnant women aged 18 years or over, with a Body Mass Index of +/=30 (kg/m^2^) and between 12 and 20 weeks gestation will be recruited. Women allocated to the control group will receive usual care and two leaflets giving advice on diet and physical activity. In addition to their usual care and the leaflets, women allocated to the intervention group will be offered to attend a weekly 1.5 hour weight management group, which combines expertise from Slimming World with clinical advice and supervision from National Health Service midwives, until 6 weeks postpartum.

Participants will be followed up at 36 weeks gestation and at 6 weeks, 6 months and 12 months postpartum. Body Mass Index at 12 months postpartum is the primary outcome. Secondary outcomes include pregnancy weight gain, quality of life, mental health, waist-hip ratio, child weight centile, admission to neonatal unit, diet, physical activity levels, pregnancy and birth complications, social support, self-regulation and self-efficacy. A cost effectiveness analysis and process evaluation will also be conducted.

**Discussion:**

This study will evaluate the effectiveness of a theory-based intervention developed for obese pregnant women. If successful the intervention will equip women with the necessary knowledge and skills to enable them to make healthier choices for themselves and their unborn child.

**Trial registration:**

Current Controlled Trials: ISRCTN25260464

Date of registration: 16^th^ April 2010.

## Background

### Obesity: the problem

The Foresight Report (2007) estimates by 2050, 50% of women could be obese and National Health Service (NHS) costs associated with obesity could be £10 billion per annum [1]. Approximately 1 in 5 women attending antenatal care in the United Kingdom (UK) are obese [2,3] and this figure is likely to increase. In Europe and the United States of America (US) between 20 and 40% of women gain more weight during pregnancy than is routinely advised [4]. Pregnancy is a significant factor in the development of obesity in women. Many women retain cumulative weight gained over several pregnancies and women with high weight gain during pregnancy retain more weight at follow-up [5-7]. Excess maternal weight gain during pregnancy is also associated with child obesity at 3 years and in adolescence [8,9]. This suggests there is potential for influencing the mother’s lifestyle and weight as well as the child’s weight.

Obesity has been linked to an increased risk of complications during pregnancy and birth including pregnancy-induced hypertension [2,10], gestational diabetes mellitus [2,11], increased emergency and elective caesarean section rates [2,10], increased induction of labour rates [2,11], venous thromboembolism [12] and increased postpartum haemorrhage [2,13]. There are also increased risks for the child including pre-term birth [2,11], shoulder dystocia [14], admission to a neonatal unit [2,13], birth defects (e.g. spina bifida, omphalocele) [14], still birth [2,13], macrosomia [2,15], fetal and neo-natal death and poor Apgar scores [16]. Consequently, the NHS costs are significantly higher in overweight and obese pregnant women compared to women in the normal weight range. Antenatal care costs may be 5–16 times higher in overweight and obese women [2,17].

### Obesity interventions

Clinicians are often uncomfortable dealing with their patients’ obesity [18,19], referral options are limited and few evidence based interventions to tackle obesity during pregnancy exist. In addition, although the Institute of Medicine (IOM) in the US has produced guidance on appropriate pregnancy weight gain for obese or overweight women this remains somewhat controversial as research evidence is limited and the guidance is based on observational data [20,21]. UK guidance is also lacking [22].

In the wider population there is evidence that lifestyle or behavioural interventions including modifications of diet and/or physical activity can help with weight loss even in the longer term [23-27]. However, interventions often have limited effectiveness, are costly and weight regain is common [23,27,28]. In the UK, the National Institute for Health and Care Excellence (NICE) has suggested that commercial weight management groups are a treatment option for obese patients [29]. Trials of commercial weight management groups have shown these approaches to be effective in the short term [30,31]. However, evidence for longer term effectiveness is lacking.

With regard to pregnant obese or overweight women, a recent large randomised controlled trial (RCT) found no impact of a lifestyle intervention on gestational weight gain (GWG) or on the proportion of women whose weight gain was below or within IOM guidance [32]. The intervention did not reduce the risk of large for gestational age infants nor did it improve maternal outcomes. However, the intervention was associated with a reduction in the risk of birth weight above 4000 g. A recent high quality meta-analysis of RCTs of interventions of diet and physical activity, alone or in combination, which included studies where women were obese, overweight and normal weight, found an overall 1.42 kg difference between intervention and control participants in GWG (in favour of the intervention group) [33]. For diet alone the difference was 3.84 kg. For interventions targeting obese or overweight women only the reduction in GWG was 2.1 kg. This review also found that reductions in pregnancy weight gain were not associated with an increased rate of small for gestational age babies. Interventions were associated with a lower risk of pre-eclampsia and shoulder dystocia and there was a trend towards a reduction in gestational diabetes, gestational hypertension and pre-term birth. However, the quality of the evidence was low for clinical outcomes as there was evidence of significant heterogeneity in the effect size, study level biases including issues with randomisation, incomplete outcome data, blinding, as well as risk of publication bias [33]. Other systematic reviews and meta-analyses have also found lower GWG from diet and physical activity interventions, but included studies often had methodological limitations including high loss to follow-up, small sample sizes and problems with blinding [34-38]. A Cochrane review of interventions to prevent excessive weight gain during pregnancy concluded that due to small effect sizes and methodological limitations of studies no intervention could be recommended for limiting excessive GWG [39].

With regards to postpartum weight loss, a recent systematic review including 12 trials indicated that a combination of diet and exercise or diet alone can help women lose weight in the postpartum period [40]. In addition, women in intervention groups were more likely to achieve a healthy weight. The authors did not find a difference between the amount of weight lost between the diet alone or diet and physical activity together. They caution that weight loss was moderate and that there were a number of methodological shortcomings in some trials. They also noted that there was much variation in the type, intensity and duration of interventions. Another systematic review also found that diet and supervised physical activity based interventions could lead to greater postpartum weight loss of 1.5 kg in the intervention compared to control group [41].

Diet and physical activity changes are key to weight loss but trials usually include other behavioural components as part of the intervention. The NICE guidance on obesity [29] and the new draft guidance on behaviour change [42,43] recommend self-monitoring and feedback, goal setting, planning and social support. Self-monitoring is important for successful behaviour change for weight loss [44]. In a meta-analysis of behaviour change interventions of physical activity and healthy eating, more effective interventions were shown to combine self-monitoring with at least one other technique derived from Control Theory (e.g. intention formation, specific goal setting) [45]. Social support is associated with improved weight loss as well as an increase in people completing treatment and maintaining weight loss [46,47]. Social support may offer benefits such as encouragement, feedback, and role modelling or peer pressure for healthy behaviours.

With regards to interventions to limit GWG, a recent meta-analysis identified that behavioural intervention components including providing information, motivational approaches, self-monitoring and rewards contingent on success were important, and using these alongside dietary interventions could be more effective [48]. Authors of this review suggest that further research is needed to identify the most effective behavioural components for limiting GWG. Another systematic review exploring lifestyle interventions which utilised goal setting approaches found that successful interventions included personalised goal setting for diet and physical activity, self-monitoring and feedback [49]. However, the authors highlighted a lack of theory in the design and evaluation and methodological problems with many studies including issues around blinding, high drop out and lack of information on intervention fidelity. Finally, a meta-analysis examining characteristics of successful interventions to reduce GWG found that diet and physical activity interventions were effective in limiting GWG. However, the authors identified that developing an understanding of the processes that lead to behaviour change and determining key behaviour change techniques is difficult because of poor reporting of the content of interventions, alongside lack of measurement of psychological determinants or behavioural outcomes. They suggest that better description of theory and the behavioural components of interventions, as well as assessing behavioural outcomes and theorised mechanisms of the effect of interventions is required [50].

The intervention being tested in this trial is a complex intervention which includes many of the effective components described above [51]. The proposed intervention is based on Social Cognitive Theory [52] and Control Theory [53] and includes techniques associated with these theories that have shown to be efficacious in changing weight related behaviours in systematic reviews and meta-analyses [26,44,45,54,55]. These include boosting self-efficacy, goal setting, modelling, encouragement, feedback and self-monitoring. Other elements of effective behaviour change such as action planning, problem solving, tailoring and social support are also central to the intervention.

### Rationale

Pregnancy is a time of change in women’s lives and is a potentially important point at which to influence women’s health behaviours as well as those of other family members [56]. Weight loss interventions with one individual can have spin-off effects on other family members [57]. Therefore, intervening with pregnant women and equipping them with the skills, knowledge and support necessary to manage their weight effectively, both during their pregnancy as well as after (thereby preventing excessive weight gain during pregnancy and retention of weight), is an important step in tackling obesity in this group.

An effective intervention would decrease obesity-related health risks for the women, reduce the risk of complications for mother and baby during childbirth and reduce health service costs. This could have a long term impact on not only the mother, but the child and other family members, resulting in far reaching public health benefits [56,57]. Although trials targeting GWG or weight loss postpartum by using advice on diet and/or physical activity have had some success [58,59], many studies have methodological problems including issues with randomisation and blinding, poor retention, incomplete follow-up data, small sample sizes, issues relating to intervention fidelity, poor description of the intervention and lack of a theoretical basis [58,60]. As such, more evidence is required. This trial seeks to address some of these methodological shortcomings, in that: it is adequately powered; it is theory-based; moderators of intervention effect are being measured; there are a number of different strategies in place to retain participants; there is a detailed process evaluation assessing issues like fidelity; and, there is a cost effectiveness analysis. As far as we are aware no RCTs of pregnancy or postpartum interventions have included an assessment of cost effectiveness. This trial will test a theory-based intervention targeting longer term postpartum weight control as well as weight gain during pregnancy.

### Aim of the study

The primary aim is to assess whether a weight management intervention for obese pregnant women is effective in reducing the women’s Body Mass Index (BMI) 12 months after giving birth.

Secondary aims include:

•to examine whether the intervention leads to lower weight gain during pregnancy;

•to assess whether the intervention leads to fewer complications during pregnancy, at birth and postnatally;

•to examine the impact of the intervention on diet, physical activity levels, health related quality of life, mental health, self-efficacy, social support and breast feeding;

•to examine the child’s weight gain;

•to examine mediators and moderators of change;

•to conduct a cost effectiveness analysis;

•to conduct a process evaluation to examine participant views, drop out, fidelity, duration of participation in the intervention and associated factors.

## Methods/design

### Ethical approval

The study will be conducted in accordance with the recommendations for physicians involved in research on human participants adopted by the 18^th^ World Medical Assembly, Helsinki 1964 and later revisions. The study has been approved by the Research Ethics Committee for Wales (Reference number 09/MRE09/58).

### Design

The study is a cluster randomised controlled trial; the maternity units, rather than individual participants, are the units of randomisation. This is to minimise risk of contamination of control participants through two potential mechanisms. Firstly, the use of aspects of the intervention with control participants by site midwives who have been trained in study procedures and secondly, the passage of information regarding the intervention between intervention and control participants who are acquainted and attend the same maternity unit.

In addition to receiving usual NHS care, all study participants will be provided with 2 study leaflets: 1) a Food Standards Association eating during pregnancy leaflet detailing foods which should be avoided during pregnancy and 2) an exercise during pregnancy leaflet detailing recommended physical activity during pregnancy and warning signs for when to stop exercise and seek medical attention. Participants attending intervention sites will also receive the HELP Study intervention which is described below. Participants attending control sites will only receive usual care and the leaflets.

### Study intervention

**Figure 1:**
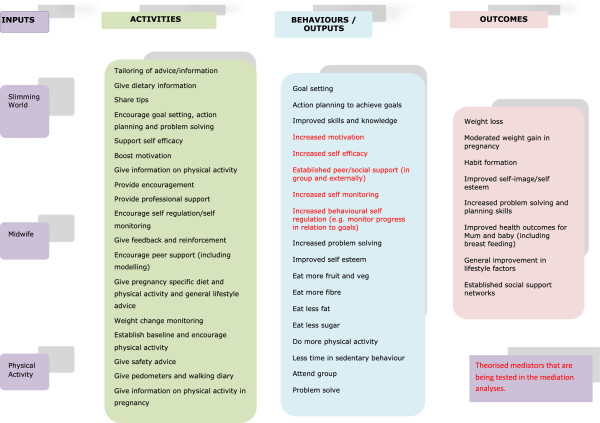
HELP Intervention Logic Model mapping the theory of the intervention.

A logic model (shown in Figure 1) describing the theory of the intervention was developed. This illustrates the key inputs, outputs/behaviours and outcomes of the intervention. Participants attending intervention sites will have the opportunity to attend free, weekly, 1.5 hour weight management group sessions from the point of recruitment (between 12 and 20 weeks gestation) up until 6 weeks postpartum. At this time point they will receive one voucher for a free Slimming World session at a ‘normal’ community group. They will also receive two intervention phone calls from the Intervention Midwife at 3 and 6 months postpartum in order to provide longer term support and encouragement. Long term intervention contact helps sustain weight loss [61] and telephone support can be effective in weight loss interventions [62]. Assuming normal gestation of between 37 and 42 weeks and depending on when women were recruited, the intervention period will be up to 56 weeks in total.

The intervention sessions will be held in NHS Antenatal Clinics and will be run jointly by an NHS midwife and a Slimming World consultant. There are four main components of the intervention group sessions: 1) healthy eating, 2) physical activity, 3) midwifery advice and 4) behavioural component.

1) The healthy eating component

Slimming World, a major UK based commercial slimming organisation has developed a flexible weight management and healthy eating programme called “Extra Easy”, which follows current UK government recommendations for a healthy diet including the “Eat Well Plate”. The diet consists of a combination of different food types: approximately 80% combined from fruit, vegetables, carbohydrates and protein; a smaller section for milk and dairy; and an allowance for foods high in fat or sugar. Other than limiting the intake of high fat or high sugar foods, it is not a ‘restrictive’ diet. Pregnant women are offered advice to encourage them to eat additional healthy extras to ensure they have adequate calcium and fibre intake. The programme utilises a “food optimising system” to encourage adherence to the healthy eating plan by considering and modifying energy density and satiety, complemented by flexibility of food options.

2) The physical activity component

An individualised physical activity programme for obese pregnant women was developed, based on The Royal College of Obstetricians and Gynaecologists guidelines for exercise in pregnancy [63]. Due to its flexibility, ease and cost effectiveness, walking is the primary focus of the programme. Women will be provided with a pedometer and walking diary in order to record daily step counts for up to seven consecutive days at various time points (baseline, 36 weeks gestation, 6 weeks postpartum, 6 months postpartum and 12 months postpartum). Women’s physical activity tends to decline as pregnancy progresses and therefore the use of pedometers is intended to act both as a motivational tool to encourage physical activity but also as a device to facilitate self-monitoring of physical activity [64]. Step count targets will be individually agreed as part of the walking intervention based on the following four study recommendations and taking into consideration government recommendations of 30 minutes of physical activity five days a week which equates to 10000 steps per day [65]. All women will be encouraged to increase their step counts gradually and as they feel able, as follows:

1 If previously sedentary, women will be advised to aim to walk for 15 minutes, three times per week, gradually increasing to 30 minutes, five times per week.

2 If previously moderately physically active, a maintenance activity plan will be negotiated based on current step counts.

3 If current step counts are greater than 10000 per day, women will be advised to continue as able within limits of comfort but not to start new modalities of physical activity.

4 Following birth, women will be encouraged to restart walking by gradually increasing daily step counts as soon as they feel able. If delivery was complicated, consultation with the women’s general practitioner (GP) or midwife will be advised prior to restarting the walking programme.

Women who are unable to complete the walking programme will be encouraged to undertake alternative recommended physical activity including swimming, aquanatal and prenatal exercise classes, as appropriate.

In order to prevent over-exertion, women will be advised to only partake in moderate physical activity and they will be asked to utilise the Borg Scale of Perceived Exertion [66] each time they do any physical activity to monitor levels of exertion. The Borg scale runs from 6 (no exertion) to 20 (maximum exertion), women will be asked to engage in activity that is ‘somewhat hard’ (around 12-14 on the scale). Information regarding warning signs to terminate exercise and when medical advice should be sought will be provided. However, for the majority of women, exercise is safe for both mother and foetus throughout pregnancy and initiating moderate exercise or continuing exercise is recommended in most pregnancies [63].

3) The Midwife component

In addition to the usual NHS midwifery care, the Intervention Midwife will be available to provide advice regarding pregnancy and lifestyle, as well as provide additional support in topics that women may be anxious about like labour choices and breast feeding. Evidence indicates that obese women are less likely than their non-obese counterparts to breast feed [67] and breast feeding is associated with reduced postpartum weight retention [68]. The study is recommending a healthy, balanced and unrestricted diet in pregnancy and therefore foetal weight should not be impaired. Any woman in the intervention group who loses a cumulative 3 kg over the pregnancy will be reviewed by the Intervention Midwife and asked to complete a 7-day food diary to confirm that she is eating a healthy amount of food. If necessary, the Intervention Midwife will refer the participant to their obstetrician. Participant safety will be the responsibility of the Intervention Midwife and any concerns will be referred to an appropriate medical practitioner as per normal protocol within the health care service.

Each session will include the following:

4) Behavioural component

•The weighing session where each woman attending will be weighed.

•New members will be welcomed and achievements of the group reviewed.

•Nutritional advice will be given and the group will discuss topics such as foods to avoid in pregnancy, sharing ideas, recipes, eating out ideas. The women will also be given access to Slimming World resources such as recipe books and magazines.

•Physical activity advice will be given and the group will review progress, share experiences, hints and tips, and discuss local activities like aquanatal. All participants will have their step targets reviewed monthly.

•Advice will be given on ailments during pregnancy such as symphysis pubis dysfunction and sciatica.

•Discussion of a ‘Topic of the week’ such as ‘eating for two’, nausea and breast feeding.

•Opportunities for one-to-one advice with the Intervention Midwife or Slimming World consultant.

Practical skills and strategies for managing behaviour change will be discussed in the groups. The Slimming World approach (http://www.slimmingworld.com/health/how-sw-works/image-therapy.aspx) provides motivational support and aims to raise self-esteem and empower members. It involves aspects of Transactional Analysis [69], Motivational Interviewing [70] and Compassionate Mind Theory [71]. The approach taken within the groups is similar to Motivational Interviewing, as it is collaborative and seeks to strengthen motivation for change, while avoiding judgment or criticism. It uses empathy, acceptance and compassion to help individuals to overcome barriers and identify goals and their own reasons or motivators to change.

In the groups there will be a level of tailoring to individuals in terms of the diet and physical activity advice and as described above, the participants will have the opportunity to discuss these as well as plans and goals individually with the Slimming World consultant or Intervention Midwife, as well as within the wider group. A number of behavioural strategies will be discussed and encouraged during the group sessions these include; self-monitoring, self-regulation, goal setting, problem solving and action planning. Within the groups different behaviours will be modelled both by the intervention staff but also by other women in the group e.g. where they have started aquanatal classes or started cooking with fresh vegetables. The intervention staff will give encouragement and feedback to the women not only on their weight but also diet and physical activity and other issues.

The key aims of the groups are to encourage goal setting, self-monitoring and behavioural self-regulation, improve motivation and boost self-efficacy and social support. These are addressed directly by the Slimming World approach. Intervention staff will also be trained by the study team on the importance of encouraging and supporting women with respect to these aims. Women will be encouraged to weigh at least weekly and to monitor and, if necessary, alter their eating and physical activity behaviours in relation to their goals. The groups seek to enhance women’s motivation by providing positive feedback and by helping them set realistic goals, problem solve and manage lapses or setbacks appropriately. It is intended that the groups will improve women’s self-efficacy by providing them with useful information and by helping them develop the necessary skills for a healthy lifestyle, but also by helping them build on success and by giving them opportunities for observing similar others succeeding (modelling) as well as providing positive feedback on progress [72]. Social support will be provided by other women within the groups as the setting facilitates sharing experiences and information, giving feedback, empathy and encouragement as well as reinforcement of behaviours which may help increase motivation. It also provides opportunities for role modelling, improved self-efficacy, instrumental support (help), appraisal (e.g. affirmation) and peer pressure for healthy behaviours.

Regular attendance at the groups will be encouraged and participants will be contacted if they miss two or more consecutive intervention group sessions, to try to foster future attendance. Intervention group sessions will be held at a convenient time to enhance attendance, usually early evening.

The Intervention Midwives and Slimming World consultants will attend a one day training workshop delivered by the study team and will receive a study manual detailing all aspects of the intervention, in order to ensure consistency in the delivery of the intervention across all sites. In addition, intervention group sessions will be observed by study team members to examine intervention fidelity across sites.

### Sample size

At the time we were developing the study we could find no systematic reviews of lifestyle interventions in pregnant women, so we based our sample size on a systematic review of interventions with obese adults, which found a mean weight loss of 7.9 kg (8.5%) during the first 6 months of interventions involving diet and exercise, after which weight was gradually regained, by 48 months a mean weight loss of 3.9 kg (4%) was maintained [28]. Results for trials which included only obese women (mean BMI+/=30 at baseline) demonstrated weight loss of a similar magnitude at 12 months [28]. In order for an individually randomised trial to have 80% power to detect a moderate effect size of 0.333 for a difference in BMI at 12 month follow-up of 1.5 kg/m^2^ (SD = 4.5), at a 5% significance level, 143 women per group would be required. Little pertinent data are available for the estimation of the intra-cluster correlation coefficient (ICC). Assuming an ICC of 0.02, if 20 maternity units were recruited across England and Wales, a variance inflation factor of 1.4 would result, so the total sample size is therefore inflated to 400 to detect the difference stated above and we would require an average of 20 women per unit. This would allow for a variance inflation factor of 1.4 (ICC = 0.02) [73]. In trials investigating weight management interventions in pregnant women, losses to follow-up range considerably from 5% to 38% [58,59,74-76], therefore we have allowed for a drop out of 30% and intend to recruit 570 women.

### Centre recruitment

Twenty maternity units across England and Wales will be recruited, ensuring a spread of different demographic areas, e.g. areas of high minority populations and low socio-economic status. All units will use electronic maternity information systems, in order to facilitate collection of outcome data and will have at least 1500 births per year. We will exclude any centre currently running a service similar to the HELP intervention.

### Participant recruitment

570 pregnant women with a Body Mass Index (BMI) of +/=30 aged 18 years or older and between 12 and 20 weeks gestation will be recruited. Potentially eligible participants will be approached at their earliest antenatal appointment by NHS midwives or researchers, who will provide an information sheet and briefly describe the study. The decision to approach women will not be made by the midwife delivering the intervention. If women are eligible and interested in participating, they will be contacted by the research midwife (local Principal Investigator (PI)) to discuss the study in greater detail and arrange a baseline home visit where informed consent and baseline measures will be taken.

The approaching midwife/researcher will provide the women with a comprehensive information sheet prior to the baseline visit and adequate time will be given for them to read the material and to ask any questions they have about the study. Women will be reminded that they retain the right to withdraw consent for participation in any aspect of the trial at any time without their routinely available NHS care being affected. Midwives/researchers will be trained in Good Clinical Practice and all study procedures. The participant’s GP, named midwife and obstetrician, if applicable, will be informed that she is taking part in the study.

A screening form will be completed to record the number of women approached about the study, eligibility, and at what stage women declined to take part in the study (e.g. when first approached or at the consent stage).

### Exclusion criteria

Women will be excluded from being recruited into the study if they:

1) are unable to understand the intervention, e.g. have insufficient understanding of spoken English;

2) have any detected pregnancy related complications e.g. multiple pregnancy, foetal anomaly, current antenatal, maternal or foetal complications, recurrent miscarriage (three or more) or previous pre-eclampsia;

3) have any previous medical complications e.g. cardiac disease, serious respiratory disease including severe asthma, diabetes mellitus, serious mental illness/psychological illness, epilepsy requiring anticonvulsant therapy or hypertension requiring treatment;

4) have nutritional complications e.g. serious physical or psychological disorders (eating disorders) or previous surgery for weight problems;

5) are involved in any other research that may affect any of the outcome measures that are being investigated in this study.

This list is not considered exhaustive. If the midwife considers that the woman has other serious complications that would affect her suitability to participate in the study, the midwife may at her discretion exclude the woman from recruitment noting on the recruitment form the reason why the woman has been excluded. If medical or obstetric complications arise while a participant is involved in the study advice will be taken from the woman’s lead obstetrician on whether she should withdraw from the intervention. If a woman is withdrawn on clinical grounds the study team will still complete follow-up if the woman is willing.

### Site randomisation

Sites will be randomised when all necessary approvals are obtained. Randomisation will be completed to give optimal balance for geographic region, maternity unit size, ethnic mix and the proportion of the maternity unit pregnant patient population with a BMI +/=30 [77-81]. A process of optimal allocation will be undertaken [79-81]. This will involve calculation of all possible allocations and a balance statistic for each one. A proportion of all allocations with the greatest degree of balance will be identified and passed to the independent statistician on the Trial Steering Committee (TSC) blinded to unit. He will randomly select a single allocation. This will then be returned to the trial statistician. The process of optimal allocation will be carried out in two blocks of ten sites with each block allocation being chosen by the independent statistician from the 25% most optimal allocations in each case. A statistician independent of the study but within the South East Wales Trials Unit will create random numbers for intervention/control arm allocation. The rest of the trial team and the clinics themselves will be informed of allocation after site recruitment.

In the event of delayed approvals for the maternity units such that it is not possible to randomise the second block of 10 sites together, minimisation will be used. A random component will be added to the minimisation algorithm using an 80% weighted randomisation. The allocations of the first block will be used to balance the remaining sites [77,78].

### Outcomes

All outcome measures are listed in Table 1 and mediator measures in Table 2. Measures were selected following a comprehensive literature search and consultation with experts in diet and physical activity. Evidence of reliability, validity and sensitivity to change were considered in the selection process. An important issue was completion time to avoid excessive respondent burden as this could affect follow-up rates. For most of the outcomes and for the mediators there was a limited choice of measures and the final choice was inevitably a compromise between evidence of good psychometric properties and the resources and time available to complete the assessments.

**Table 1:** Measurement of Outcomes

**Outcomes**	**Measure**	When*
**Primary outcome**		
Maternal weight expressed as BMI relative to height measured at baseline	Calibrated adult scales & stadiometer	B, 36w, 6p, 6 m, 12 m
**Secondary outcomes**		
Antenatal and birth complications**	Routinely collected data held in patient records	Birth
Pregnancy weight gain	Calibrated adult scales	B, 36w
Waist circumference and waist-hip ratio	Measuring tape	12 m
Child weight centile (adjusted for birth weight and age)	Calibrated baby scales and measuring tape	Birth, 6p, 6 m, 12 m
Admission to neonatal unit	Patient records	Birth
General mental health	General Health Questionnaire (GHQ) 12 [82]	B, 36w, 6p, 6 m, 12 m
Breast feeding intentions	Study-developed questions	36w
Breast feeding behaviour and weaning	Study-developed questions	6p, 6 m, 12 m
Self-reported physical activity	7 Day Physical Activity Recall (7 Day PAR) [83-85]	B, 36w, 6p, 6 m, 12 m
Diet	Dietary Instrument for Nutrition Education (DINE) [86] (plus additional questions on fruit and vegetables, sugar, sweets)	B, 36w, 6p, 6 m, 12 m
Alcohol	Alcohol Use Disorders Identification Test-Consumption (AUDIT-C) [87]	B, 36w, 6p, 6 m, 12 m
Smoking	Study-developed questions	B, 36w, 6p, 6 m, 12 m
Costs	Participant resource use	B, 36w, 6p, 6 m, 12 m
Health related quality of life	EQ-5D (including visual analogue scale) [88]	B, 36w, 6p, 6 m, 12 m

*B = baseline; 36w = 36 weeks gestation; 6p = 6 weeks postpartum; 6 m = 6 months postpartum; 12 m =12 months postpartum.

**gestational diabetes; pre-eclampsia; thrombosis; proportion staying within IOM guidance on weight gain in pregnancy [20]; form of pain relief; birth delivery mode; gestation at delivery; induction of labour; shoulder dystocia, 3^rd^/4^th^ degree perineal tear; postpartum bleeding or thrombosis; Apgar scores.

**Table 2:** Measurement of Mediators

**Mediators**	**Measure**	**When**
Social support	Social Support Exercise and Eating Habits Scales [89] (plus intervention specific social support questions)	B, 36w, 6 m, 12 m
Self-efficacy	Weight Efficacy Lifestyle Scale [90] and Multidimensional Self efficacy for Exercise Scale [91,92]	B, 36w, 6 m, 12 m
Self-regulation	Shortened Self-Regulation Questionnaire [93]	B, 36w, 6 m, 12 m
Motivation	Treatment Self-Regulation Questionnaire (for diet and physical activity) [94]	B, 36w, 6 m, 12 m

(B = baseline; 36w = 36 weeks gestation; 6 m = 6 months postpartum; 12 m =12 months postpartum).

The primary outcome is maternal BMI at 12 months postpartum. Secondary outcomes will include investigation into the impact of the intervention on gestational weight gain, the child’s weight gain, complications during pregnancy, at birth and postnatally, diet, physical activity levels, health related quality of life, mental health and breast feeding intentions. All staff collecting data will be trained in administering the different outcome measures as well as accurately measuring weight, height and waist and hip circumference. Height will be measured once at baseline and used for all BMI calculations.

Information on both adverse events (AE) and serious adverse events (SAE) will be collected in the study. Trial sites, participants’ GPs and intervention staff are responsible for reporting AEs and SAEs. They may also be reported by participants and by staff completing follow-up. In this trial cohort the following are expected to occur: hospitalisation for normal birth or any antenatal, perinatal or postnatal complications, termination of pregnancy for foetal anomaly and hospitalisation for postnatal depression. Rates of AEs and SAEs are likely to be higher in this group of obese women than the normal population of pregnant women. There are no SAEs expected to be related to the study intervention.

### Follow-up & drop out

Baseline data will be collected by local PIs. Follow-up data will be collected by local PIs or research staff in each centre, or network research staff. For units in Wales this will be the Clinical Studies Officers employed by National Institute for Social Care and Health Research Clinical Research Collaboration (NISCHR CRC), in England the research nurses employed by the Comprehensive Local Research Networks (CLRN). The follow-up visits will be completed in the participants’ home or at a location of the participants’ choice. The follow-up appointments are timed to occur at important milestones both pre and postnatally: 36 weeks gestation, 6 weeks postpartum, 6 months postpartum and 12 months postpartum.

Every effort will be made to reduce loss to follow-up: women will be visited at a convenient location of their choice for all follow-up appointments. In order to improve response rates, participants will be contacted to rearrange any missed follow-up appointments, participant-nominated contacts will be collected at baseline in order to facilitate contact at follow-up appointments, participants will be posted study updates in the form of newsletters and follow-up calendars, and each participant will be provided with a £10 high street voucher as a thank you for completing each follow-up. A HELP study website will be used to provide study updates (http://medicine.cf.ac.uk/help-study/), and a minimum dataset will be developed to collect follow-up data by telephone for those participants who are unwilling or unable to meet with the researcher.

In order to prevent resentful demoralisation in the control group, each of these women will be offered 12 weeks normal community-based Slimming World sessions free of charge after her 12 month follow-up is complete. Any woman who undergoes a miscarriage, stillbirth, neonatal death or termination of pregnancy will be given an open option whether or not they wish to continue participation in the study.

All site study staff including the Intervention Midwife and Slimming World consultant will be visited by the research team and updated via regular contact and newsletters in order to encourage continued enthusiasm. In addition, to prevent disappointment following randomisation in the control sites, if results from the study prove positive units allocated to the control group will then be offered training for their midwives in the intervention.

### Process evaluation

A process evaluation will be conducted in line with the framework suggested by Steckler and Linnan [95]. This evaluation will utilise both qualitative and quantitative data including data taken from focus groups, interviews, site intervention group observations, session summaries. The process evaluation model will include assessment of eight components; these are context, reach, exposure, fidelity, recruitment, retention, contamination and theory testing. The definition of some of these elements is less clear than others and there is some overlap between concepts, so these are defined here as used in this study. “Context” includes information relating to different aspects of the context that the intervention was delivered in. This was explored by addressing who delivered the intervention and where it was delivered. The broader context was considered in the qualitative work and includes data on circumstances, skills, resources and attitudes that may influence intervention effectiveness. “Reach” is defined as the extent to which the target audience is reached by the intervention as well as whether the intervention had ‘spillover’ effects on other people not recruited in the trial. We were interested in exploring whether it had any impact on the family and friends of the participants. “Exposure” is defined as whether the participants received the different elements of the intervention and whether the participants implemented the different elements as intended. “Fidelity” is defined as the degree to which the Intervention Midwives and Slimming World consultants delivered the intervention as intended.

We will assess study attrition by intervention or control group as well as by site. We will compare those dropping out with those remaining in the trial in terms of demographics. We will attempt to obtain reasons for dropout where possible and record these. Finally, we will assess potential contamination between groups through the interviews and focus groups as well as by obtaining details of all other services or interventions that control group participants accessed. Table 3 shows the key sources of information used to explore the eight components of the process evaluation.

**Table 3:** Process Evaluation Elements

**Process evaluation component**	**Assessment**
Context	• Data collected on a site proforma detailing site demographics, ethnicity, size, services delivered etc.
	• Data on those delivering the intervention
	• Data from two site observations completed at different time points in the intervention delivery period using a structured observation guide.
	• Contextual issues explored in the staff focus groups and participant interviews
Reach	• Attendance at the group sessions
	• Comparison of characteristics of those attending the intervention with those not attending
	• Reach explored in the staff focus groups and participant interviews
Exposure	• Number of group sessions delivered
	• Data from group session summary forms which describe those attending and the content/timings of sessions
	• Data from site observations (two per site)
	• Attendance at group sessions
	• Exposure and attendance explored in the staff focus groups and participant interviews
	• Data gathered on use of pedometers, step targets and walking diary completion
Fidelity	• Data from site observations (two per site)
	• Data from group session summary forms which describes how the intervention was implemented at each session
	• Fidelity explored in the staff focus groups and participant interviews
Recruitment	• Comparison of demographics of sites recruited
	• Recruitment rates compared across sites in terms of how many recruited, who is recruited and also how quickly people are recruited
	• Comparison of potentially eligible women with those recruited using data from case report forms and screening forms
	• Recruitment issues explored in the staff focus groups and participant interviews
Retention	• Dropout by trial arm
	• Dropout by site
	• Comparison of demographics of those dropping out with those remaining
Contamination	• Participants asked what other services control group utilised in case report forms
	• Contamination explored in the staff focus groups and participant interviews
Theory testing	• Mediation analyses using questionnaire data
	• Theoretical mediators explored in the staff focus groups and participant interviews

A key method for assessing these components is via the qualitative data collection i.e. qualitative interviews with participants and focus groups with the staff delivering the intervention. Semi-structured interviews will be completed with approximately 30 participants from the intervention group, purposively sampled across sites according to attendance levels at the intervention group sessions and whether they lost weight or not. The interviews will be carried out at the end of the intervention period (at approximately 6 months postpartum) and at the end of the study (at approximately 12 months postpartum). We will explore the participants’ views of the intervention, barriers and facilitators, the impact of life events on adherence to the intervention, importance of social support, strategies, coping mechanisms and responses to relapses. We will interview a small sample of participants who drop out of the intervention but who are willing to be interviewed, to establish their views of the intervention and reasons for discontinuing. We will also conduct brief interviews with a sample of around 15 women from the control group about taking part in the study and any lifestyle changes they made both during and after pregnancy. The interviews will continue until data saturation is reached.

Three intervention staff focus groups will be completed and will explore the intervention components, the delivery of the intervention, intervention fidelity, participant adherence to the intervention, the recruitment process, perceived challenges or barriers in implementing the intervention and potential improvements to the intervention or the training.

With regards to theory testing we developed a logic model (this is shown in Figure 1) to explain the processes by which the intervention brings about change and we plan to test the theory of our intervention via mediation analyses as well as through other aspects of the process evaluation including participant interviews. Potential mediators including self-regulation, intrinsic motivation, self-efficacy and social support will be assessed. The analyses will identify both the extent to which the intervention was successful at changing these mediators and the extent to which mediator change was associated with change in BMI. Potential moderators of intervention effect will be examined including demographics, ethnicity, parity, mental health (also an outcome), smoking status and weight loss history.

### Economic evaluation

The main evaluation will be a cost utility analysis assessing between group differences in total costs against differences in Quality Adjusted Life Years (QALY) derived from the EQ-5D quality of life instrument [88]. This approach is preferred by the National Institute for Health and Care Excellence (NICE) for the economic evaluation of NHS interventions as resulting cost utility estimates can be compared across unrelated health care interventions (http://www.NICE.org.uk). However, as a generic measure, EQ-5D may not be sufficiently sensitive to capture small changes in health-related quality of life in essentially healthy participants. A secondary cost effectiveness analysis will therefore be undertaken with BMI as the effectiveness measure. Both analyses will be done from an NHS perspective but as the HELP intervention might substitute for other weight control interventions, patient borne costs will also be assessed but will be reported separately.

Resources for training intervention midwives and delivery of the intervention will be recorded prospectively in relevant units and valued using standard methods [96]. Participants’ use of NHS resources will be collected by questionnaire from women in both arms of the trial at baseline and all follow-up points specified above and similarly valued. The questionnaire will also record payments for non-NHS weight loss/maintenance activities and will include the EQ-5D questionnaire [88].

### Analysis

#### Quantitative analysis

The main analysis will be by intention to treat and will compare the primary outcome of BMI at 12 months postpartum in the intervention and control groups. Multilevel modelling will be used to account for clustering within antenatal unit and individual effects. A two level linear regression model will include baseline BMI (measured at recruitment) as a covariate. Both levels will be considered ‘random effects’ i.e. patients and units are drawn randomly from a larger population of patients and units. Cluster level variables include those used to balance the randomisation: antenatal unit size; proportion of women with BMI +/=30, geographic location and ethnic mix.

For the BMI data, positively skew distributed form is anticipated and will be checked prior to analysis. Log transformation will be considered, not only to deal with the non-normality but to allow interpretation of differences between arms in percentage terms. To further aid clinical interpretation of the intervention effect, analysis of log transformed weight at 12 months postpartum will also be considered, with baseline log weight and log height as covariates. The results can then be expressed in terms of BMI or weight along with a 95% confidence interval. The intra-cluster correlation (ICC) for the primary outcome will be calculated and reported.

Intention to treat analysis will be used for all secondary outcomes. Analysis of secondary outcomes will also use multilevel modelling incorporating baseline scores as covariates where appropriate. Two level linear regression models will be used for outcomes such as pregnancy weight gain and waist-to hip ratio and validated questionnaire scores, while logistic models will be used for clinical event outcomes. 95% confidence intervals for the intervention effect will be calculated. The ICC for each secondary outcome will be calculated and reported.

The impact of individual demographic factors as well as theoretical mediators (self-efficacy, social support, intrinsic motivation and self-regulation) on the intervention effect using interaction terms included in the main analysis models will be examined. Individual demographic variables include age, ethnicity, smoking status, previous weight loss history, psychological wellbeing and social class. We also intend to carry out tests for mediator variables [97].

As well as examining number of sessions attended, patterns of missed sessions and compliance with the intervention will also be explored. A complier average causal effect (CACE) analysis will be carried out for the primary outcome to assess the effect of the intervention in those who complied [97,98]. An investigation of required minimum dose of intervention will be carried out. A per- protocol analysis will include only those participants in each arm that received treatment as randomised excluding those in the control arm attending weight loss groups.

No formal subgroup analyses are planned. However, exploratory analyses of the impact of social class, parity, ethnicity (if numbers permit), smoking status and initial BMI on the effect of the intervention will be carried out. This will be achieved by fitting a subgroup by randomised group interaction term to the multilevel model.

If the proportion of missing primary data is substantial (more than 10%) a series of sensitivity analyses will be carried out to determine the likely effects of missing data [99-101]. The intention is to use multiple imputation to generate complete datasets for analysis. Imputation models will include those variables in the analytic model plus any additional variables associated with missingness and outcome. Self-reported weight (from the minimum dataset) and Slimming World session weight data may be used to replace missing weights where appropriate in secondary analyses. Further sensitivity analyses may be carried out to examine the effects of removing women who are known to be pregnant at the 12 month postpartum follow-up, as well as those who have recently given birth to a second baby. The assumptions of all models used for primary and secondary analysis will be checked.

Short and long term effects of the intervention can be examined using repeated measures analysis of intermediate weight measurements. The difference in weight at 6 months postpartum will also be examined. The proportion of participants who lost 5% of their weight (weight at 6 months postpartum compared to baseline) will be calculated for each arm. The difference between groups will be examined to identify if they maintained that loss. The association between subsets of clinical outcomes will also be investigated and a total count of all clinical outcome events will be calculated and compared between trial arms. Individuals lost to follow-up will be compared to those who complete follow-up to identify potential sample bias.

#### Qualitative analysis

Interviews and focus groups will be audio recorded, transcribed and checked by the researcher. Standard thematic analysis techniques will be employed. Transcripts will be closely examined to identify themes and categories [102]. Codes will be applied to these broad themes which will then be broken down further into sub-codes. Agreement on concepts and coding will be sought between members of the research team to ensure reliability. Commonly expressed themes will be identified as well as unusual cases. 20% of the data will be coded separately by two team members to check reliability of the coding process. Interviewing will be iterative; where new themes emerge they will be incorporated into the interviews and focus groups. Thematic analysis will be supported by qualitative analysis software (NVIVO).

#### Economic analysis

As training can be regarded as an investment producing a flow of benefits over time, training costs will be amortised and expressed in equivalent annual cost terms. Costs of delivering the intervention, including an element for training, will be apportioned to the intervention group. Mean differential costs between intervention and control groups will be estimated. As cost data are often skewed, tests for normality will be carried out and if data are not normally distributed non-parametric analyses will be used to carry out the comparison of costs between the two arms of the trial. Economic comparisons between the two study arms will take account of the cluster nature of the data.

Results of the cost utility analysis will be reported in the form of an incremental cost utility ratio (incremental cost/QALY). A series of one-way sensitivity analyses will assess how sensitive results are to changes in key assumptions. Probabilistic sensitivity analyses will be used to quantify uncertainty around the estimates and cost effectiveness acceptability curves will show the probability of the intervention having an incremental cost utility ratio below a range of acceptability thresholds [103].

In the secondary analysis, cost effectiveness will be assessed using BMI as the effectiveness measure. Unless the intervention is shown to be dominant (lower costs greater effect) the resulting incremental cost effectiveness ratio (incremental cost per unit difference in BMI) can be compared with that of other weight management programmes delivered to pregnant women.

Exploratory work will be carried out to model the medium term effect of the intervention bearing in mind the high degree of uncertainty in long term weight patterns particularly among the obese [104].

## Discussion

This trial will evaluate the effectiveness of a theory-based intervention for obese pregnant women, which combines dietary expertise from Slimming World, physical activity, and clinical advice and supervision from midwives. The intervention aims to provide support to enhance motivation and equip women with the necessary knowledge and skills to enable them to make healthier choices and control their weight gain during pregnancy as well as maintain a healthy lifestyle postpartum through healthy eating and physical activity.

The study is novel as, to our knowledge, no RCTs of pregnancy or postpartum weight control interventions have included an assessment of cost effectiveness and there are no published trials of diet and physical activity interventions that run through pregnancy and into the postpartum period [34]. In addition, few trials have explicitly described the theoretical basis of the intervention or measured the psychological mediators of the effect.

The cluster design was chosen to avoid the risk of contamination, because midwives trained in the use of the intervention could potentially use aspects with control participants and pregnant women resident in the same area often know one other and could share study information. Also in order to run effective groups the cluster design is superior in terms of recruiting sufficient numbers to ensure sessions can be delivered locally.

The study incorporates economic and process evaluations as well as explicit testing of the theory of the intervention. The process evaluation will allow us to explore the impact of different aspects of the intervention and if the trial does not show an effect it will allow us to explore possible reasons for this.

### Protecting against bias

Staff in maternity units who volunteer for the study are likely to be motivated in favour of the intervention, which may result in disappointment in those subsequently allocated to the control group. In order to avoid differential dropout between the experimental and control groups, we will offer the maternity units in the control group the opportunity to complete the training programme after the follow-up period, should the intervention prove to be successful. Careful characterisation of the participating sites, clinicians and patients will be undertaken to judge the external validity of the study findings.

Outcome data will be collected by PIs or trained researchers allied to the project. Due to the nature of the study, it will be difficult for researchers collecting outcome data to be blinded to the allocation of the women; however no staff involved in delivering the intervention will collect follow-up data.

The findings of this study will advance current knowledge in this field, both in terms of weight management interventions for obese pregnant women as well as behaviour change theory. If the trial is successful, this could alter the management of obese pregnant women within the NHS. Potential outcomes of the intervention may include fewer complications in pregnancy and postpartum for both mother and baby as well as less traumatic deliveries. Improvements in the women’s physical and psychological health and self-esteem may also result from attendance at the intervention group sessions, and from the physical activity aspect of the intervention, independent of any weight loss. Benefits to the women may be long lasting. There is evidence that many women retain weight gained during pregnancy. If this intervention is successful this may impact on cumulative obesity developing over several pregnancies. Women will also benefit from expanded healthcare choices (e.g. midwife as opposed to consultant led care) in subsequent pregnancies, if a healthy lifestyle leads to a BMI within normal limits.

### Conclusions

Obesity in pregnancy is linked to poor health and increased NHS costs. This intervention could potentially have an impact on the women taking part during their current pregnancy but it could also equip them with weight management and healthy lifestyle skills they can use in the future. Benefits to public health could be far reaching; pregnancy is a time of significant change within a family at which women who could benefit from weight control are accessible and may be readily motivated, and any change to lifestyle could influence families’ behaviour in the longer term.

## Abbreviations

AE: Adverse event; BMI: Body mass index; CACE: Complier average causal effect; CLRN: Comprehensive local research networks; DINE: Dietary instrument for nutrition education; GP: General practitioner; GHQ: General Health questionnaire; GWG: Gestational weight gain; HELP: Healthy eating and lifestyle in pregnancy; ICC: Intra-cluster correlation; IOM: Institute of medicine; NHS: National Health service; NICE: National Institute for Health and Care Excellence; NISCHR CRC: National Institute for Social Care and Health Research Clinical Research Collaboration; 7 Day PAR: 7 Day Physical Activity Recall; PI: Principal investigator; QALY: Quality adjusted life years; RCT: Randomised controlled trial; SAE: Serious adverse event; TSC: Trial steering committee; UK: United Kingdom; US: United States of America.

## Competing interests

Amanda Avery has an academic post at the University of Nottingham but also works part-time for Slimming World. Slimming World have provided some of the intervention costs for the study, however neither Amanda Avery or Slimming World will have access to the study data or will be involved in the data collection or analyses of the study. The other authors declare that they have no competing interests in relation to this study.

## Authors’ contributions

Dr. EJ led the writing of this manuscript, contributed to the protocol and intervention development and managed the trial. Dr SAS is the Chief Investigator she led the study design, wrote the original protocol and led the trial implementation. DMC completed the qualitative aspects of the trial as well as the data management. KJ led the pilot study, assisted with study design and advised on the clinical aspects of the study. Professor DC contributed to study design and led the health economics component and Dr ML assisted with the health economics component. DD assisted with study design and advised on the dietary component of the intervention. Dr RP assisted with study design and led the statistical component of the study. Professor RN assisted with study design and advised on the statistical component of the study. Dr MB assisted with study design and the design of the physical activity component of the intervention. Dr EO-J assisted with study design and advised on trial management procedures of the study. Dr NW advised on the clinical and scientific aspects of the study design. AA contributed to intervention design and advised on different aspects of the protocol. All authors contributed to and commented on the different versions of this paper. All authors read and approved the final manuscript.

## Pre-publication history

The pre-publication history for this paper can be accessed here:

http://www.biomedcentral.com/1471-2458/14/439/prepub
